# Attentional processes in typically developing children as revealed using brain event-related potentials and their source localization in Attention Network Test

**DOI:** 10.1038/s41598-018-36947-3

**Published:** 2019-02-27

**Authors:** Praghajieeth Raajhen Santhana Gopalan, Otto Loberg, Jarmo Arvid Hämäläinen, Paavo H. T. Leppänen

**Affiliations:** University of Jyväskylä, Department of Psychology, Jyväskylä, 40014 Finland

## Abstract

Attention-related processes include three functional sub-components: alerting, orienting, and inhibition. We investigated these components using EEG-based, brain event-related potentials and their neuronal source activations during the Attention Network Test in typically developing school-aged children. Participants were asked to detect the swimming direction of the centre fish in a group of five fish. The target stimulus was either preceded by a cue (centre, double, or spatial) or no cue. An EEG using 128 electrodes was recorded for 83 children aged 12–13 years. RTs showed significant effects across all three sub-components of attention. Alerting and orienting (responses to double vs non-cued target stimulus and spatially vs centre-cued target stimulus, respectively) resulted in larger N1 amplitude, whereas inhibition (responses to incongruent vs congruent target stimulus) resulted in larger P3 amplitude. Neuronal source activation for the alerting effect was localized in the right anterior temporal and bilateral occipital lobes, for the orienting effect bilaterally in the occipital lobe, and for the inhibition effect in the medial prefrontal cortex and left anterior temporal lobe. Neuronal sources of ERPs revealed that sub-processes related to the attention network are different in children as compared to earlier adult fMRI studies, which was not evident from scalp ERPs.

## Introduction

Visual attention identifies and selects information that is relevant to ongoing behaviour, and ignores information that is irrelevant^1^. Several studies have described the development of sub-components of visual attention in children and adults using behavioural paradigms; these studies have also mapped the time course of brain activity across brain areas related to attention networks^2–6^. However, to the best of our knowledge, there are no electroencephalography (EEG) investigations of both event-related potentials (ERPs) and their underlying neuronal sources related to attention networks in children in a single study. In this study, we examined reaction times (RTs), brain ERPs, and neuronal sources associated with attention network sub-components using a modified attention network test (ANT)^7^ in typically developing school-aged children.

ANT is an experimental task which measures three sub-components of attention: alerting, orienting, and inhibition^7,8^. ANT^9^ is a combination of Posner’s cued detection^10^ and Eriksen’s flanker task^11^. In this test, participants are asked to detect the direction of a middle target item out of a group of five items, often an arrow^5^ or, in studies with children, a fish^6^. The target stimulus is either preceded by a cue (centre, double, or spatial) or without a cue (no cue), in order to manipulate the alerting and orienting sub-components of attention^12^. In addition, the direction of the target item can either be congruent or incongruent in relation to the flanker items, thereby manipulating the inhibition sub-component of attention.

Alerting in the framework of attention refers to achieving and maintaining a state of sensitivity to incoming stimulus^8^. Alerting effects can be measured by the difference in RTs to a target stimulus, with a cue versus without a cue. Previous studies showed that a warning cue helps increase alertness and decrease RTs to the target stimulus^5,12,13^.

This alerting effect is also observed in children, although their RTs vary with age and are slower than those of adults. For example, a previous study found that in response to a target with an alerting cue, five-year-old children tended to have longer RTs than seven-year-old children^14^. Similarly, RTs for the ANT in both 10-year-old children and adults have shown significant improvements in behavioural alerting scores as age increases^12^.

The functioning of the attentional processes at the brain level has been investigated using ERPs in both children^6,15^ and adults^5,16^. The behavioural effects for alerting (visual cue vs no cue) are accompanied by modulations to the posterior visual N1 amplitude at 100–280 ms for the target stimulus^5,16–19^. Previous research showed that both hemispheres exhibit alerting effects, but that they may be stronger in the right hemisphere^7,20^. Generally, in adults, N1 amplitude over the occipital and parietal regions reflects visual processing of target stimulus properties, and is modulated by cue conditions^5,21,22^. However, to the best of our knowledge, there are no studies that employ visual cue manipulation of the N1 alerting effect in typically developing children.

Adult functional magnetic resonance imaging (fMRI) studies have shown that the alerting network is associated with increased activity in the thalamus, temporal parietal junction (TPJ), and prefrontal cortex^13,22^. Recently, a slightly modified experimental design produced results that revealed additional brain areas being activated by alerting in the anterior cingulate cortex (ACC), frontal eye fields (FEF), occipital, and visual areas^23^. These findings imply that the activation of these areas is associated with response preparation and anticipation based on the visual warning cue^23^. The alerting network in children shows neuronal activity in the middle occipital cortex, which extends towards the right superior temporal gyrus. It is suggested that the differences in neural correlates of alerting effects between children and adults are due to maturational changes in neuronal network organization that occur during development^13^.

The second sub-component of interest, orientation, is associated with spatial selection. Spatial orienting has three distinct sub-functions: the engagement of visual attention to a particular stimulus, the disengagement of visual attention from a stimulus, and the shifting of visual attention from one stimulus to another^7^. Like alerting effects, orienting effects can be measured by a difference in RTs between centre-cued and spatially-cued target stimuli. This ability to shift attention between stimuli tends to improve between 5 and 14 years of age, and improves further into adulthood, thereby showing progressive development in orienting effect over an extended age period^12,24,25^.

Orientation of attention to a cued target stimulus location enhances the N1 ERP amplitude between 110 ms and 280 ms in children^26^ and adults^16,19^. Consistently, studies on adults have shown that spatially-cued target stimuli elicit larger N1 amplitude than centre-cued target stimuli, which suggests stronger engagement and lasting effects for the spatial cue with regard to the target stimulus^16,27^. Further, studies have suggested that both the right and left hemispheres of the brain are involved in orienting attention^20^. In addition, the N1 enhancement for orienting to the cued target stimulus location has been observed over occipital and parieto-occipital scalp areas^5^, which is similar to the alerting effects described above.

The topography of ERP responses that reflect the orientation effect is partially consistent with the network nodes found in adult fMRI studies, particularly at the TPJ. fMRI studies related to orienting also found other regions, such as the bilateral superior parietal lobe, FEFs, pulvinar, and superior colliculus^13,22,23^. Further, the pulvinar has also been shown to be active during the engagement of visual attention at a particular stimulus in specific spatial location; the posterior parietal lobe is involved in the disengagement of visual attention from a stimulus, while the superior colliculus along with the FEFs and interparietal sulcus are related to the ability to shift visual attention from one stimulus to another^2,7^. A study examining the orienting network in children found responses in the superior frontal gyrus and bilaterally in the occipital cortex^13^.

The third sub-component of interest, inhibition, includes mechanisms for resolving conflicts, detecting errors, and selecting actions in response to target stimuli^8^. The inhibition effect, as it relates to conflict resolution, can be demonstrated by the RT difference between incongruent and congruent target stimuli^9^. Previous studies on children^13,14^ and adults^5,9^ have suggested that such conflicts could increase inhibition of competing visual information and produce interference to response selection.

As with the other sub-components of attention, children show longer RTs than adults when resolving incongruence, but children’s RTs improve from 4 to 7 years of age^28^. However, the development of inhibition does not appear to be linear, as there is a larger improvement in RTs from 5 to 10 years of age, while there is a smaller change or no difference between 10-year-olds and adults^12,29^.

Inhibition effects can be observed as a modulation of the P3 segment of the ERP amplitude^5,6,15,16^. In the context of the ANT, P3 reflects neural activity related to the processing of cueing information and response control (motor selection)^30^. The time window of P3 is 300–650 ms from target stimulus onset^5,6,16^, with a maximal amplitude typically over the centro-parietal scalp area^5,31^. In the ANT, target stimulus-generated P3 amplitudes tend to appear at later latencies in children (10 years old)^6^ as compared to adults^16^, thereby reflecting a developmental trend in the evaluation of target direction^32^ and suggesting a rather late development of inhibitory processes^33^.

fMRI sources of an inhibition network have been examined in both children^13,15,34^ and adults^22,23,35^. In adults, inhibition appears to include activation of the right ACC, bilateral precentral gyrus, intraparietal sulcus, anterior insular cortex, FEFs, as well as right, middle, and left inferior occipital corteces^23^. In particular, the ACC plays an important role in resolving conflicts^4^. Two theories based on computational models suggest that the ACC is engaged in monitoring conflict, and the lateral frontal areas are involved in resolving the conflict^36^. Large developmental differences exist between children and adults during effective response inhibition^34^. In children (aged 8–12 years), inhibition processes involve the right superior temporal gyrus, bilateral parietal cortex, bilateral occipital cortex, and premotor cortex; however, these processes show less prefrontal cortex activation (inferior and medial frontal gyrus) as compared to that in adults^13,34^. These results suggest an immature development, particularly in the frontal area, in children for the inhibition network^34,37^.

Previous studies that employed the ANT used EEG^5,6,27^ and fMRI^13,15,22,23^ to demonstrate the time course of attention-related brain activations. Yet, ERP studies on attentional processes utilizing attention network test (ANT) paradigm in school-aged children are rare. Although orienting and inhibition sub-processes have been examined before in children, there is a lack of knowledge on target stimulus N1 in children in the form of a visual alerting cue (double vs non-cued). Further investigation of neuronal sources using high-temporal resolution EEG in typically developing children to identify the brain areas associated with the three attentional sub-processes would help us to understand the time course of activations in the different brain regions involved in the attention network. Our study could provide a reference point, for example, to evaluate attention network sub-components related to linear text reading^38^ in children at the brain level, and how this relationship is different in children with attentional problems^6,39^.

Based on previous EEG-based studies^5,6,15,16^, we expect that alerting and orienting in children would produce larger N1 amplitude for double-cued vs non-cued target stimulus and spatially-cued vs centre-cued target stimulus, respectively, which are potentially associated with efficient processing of stimulus^5^; we also expect that inhibition would produce larger P3 amplitude for a congruent target stimulus, which is associated with the evaluation of target stimulus^21^. Further, based on previous fMRI studies^13,22,23^, we also expect that alerting effects would modulate activity in the bilateral occipital lobe, right temporal lobe, FEFs, and prefrontal cortex; orienting effects would modulate activity in the bilateral occipital, parietal, and frontal lobes, and FEFs; and inhibition effects^13,15,22,23,34,35^ would modulate activity in the bilateral occipital and parietal lobes, right temporal lobe, FEFs, and the medial and prefrontal cortices.

In this study, we investigated the modulation of the N1 amplitude to the target stimulus for both the alerting and orienting networks, and the modulation of target stimulus P3 for the inhibition network in typically developing children aged between 12 and 13 years as part of a larger project^38^ (eSeek—Internet and Learning Difficulties: A multidisciplinary approach for understanding reading in new media). We used spatio-temporal topographic maps and the classical LORETA analysis recursively applied (CLARA^40^) distribution model to separately identify the activation of neuronal sources in these three networks. We also performed dipole source modelling using spatial constraints provided by CLARA solutions.

## Results

### Reaction-time performance

#### **Event-related potentials**

**Figure 1:**
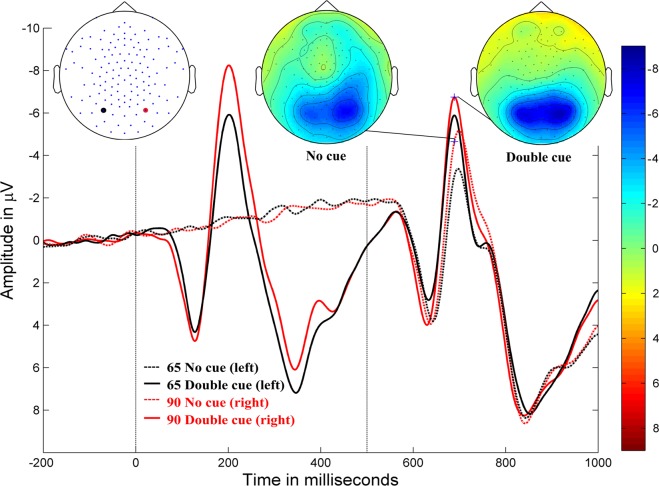
Alerting. Grand-averaged ERP waveforms for the double-cued stimulus (solid lines) and non-cued stimulus (dotted lines) for posterior electrodes (90, red, right hemisphere; 65, black, left hemisphere) in typically developing children (negativity up). Cue onset is at 0 ms and target stimulus onset is at 500 ms. Amplitude topographies for double-cued and non-cued target stimuli at 689 ms (i.e., 189 ms after target stimulus onset).

**Figure 2:**
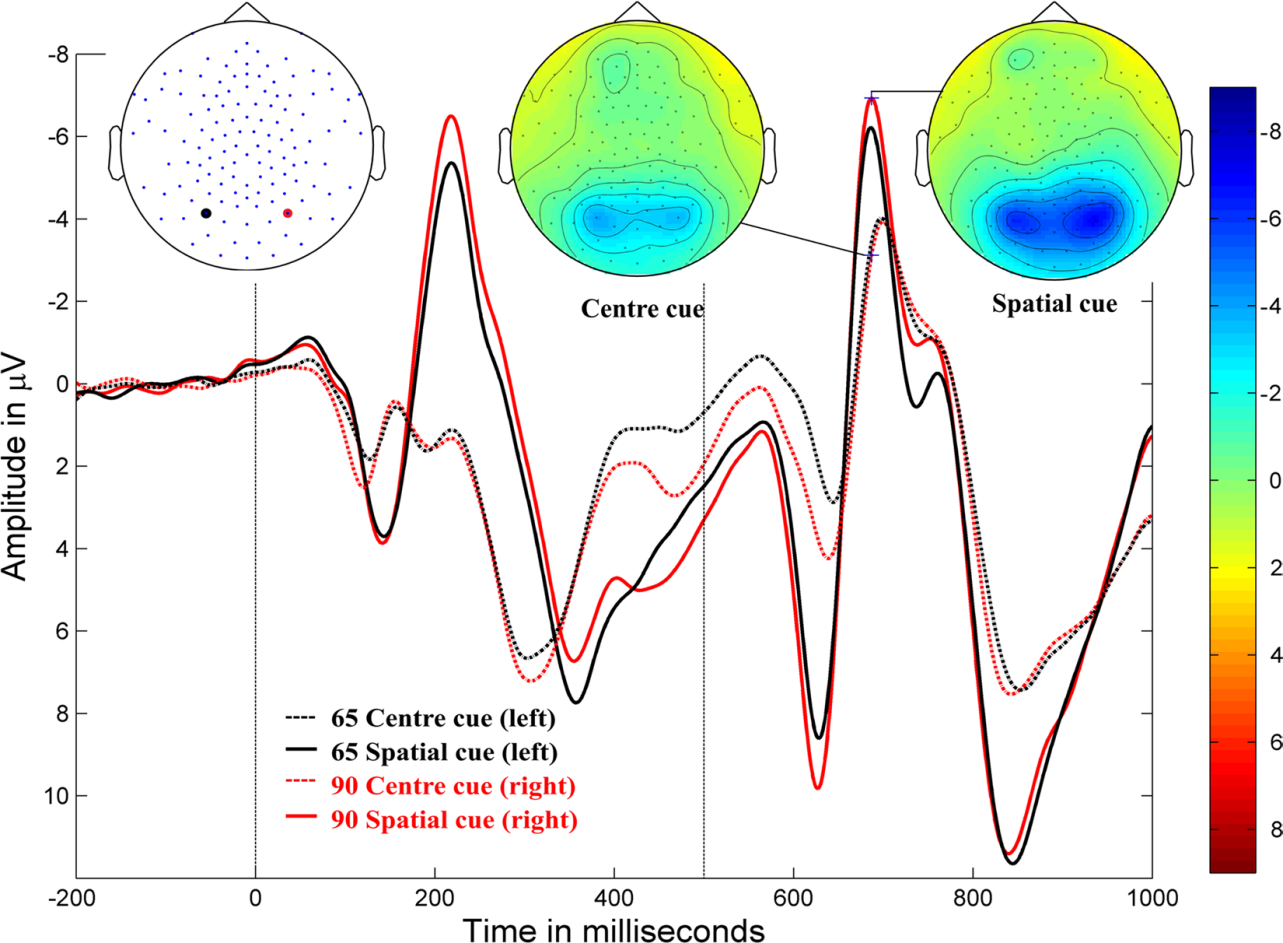
Orienting. Grand-averaged ERP waveforms for the spatially-cued stimulus (solid lines) and centre-cued stimulus (dotted lines) at posterior electrodes (90, red, right hemisphere; 65, black, left hemisphere) in typically developing children (negativity up). Cue onset at 0 ms and target stimulus onset at 500 ms. Amplitude topographies for spatially-cued and centre-cued target stimuli at 686 ms (i.e., 186 ms after target stimulus onset).

**Figure 3:**
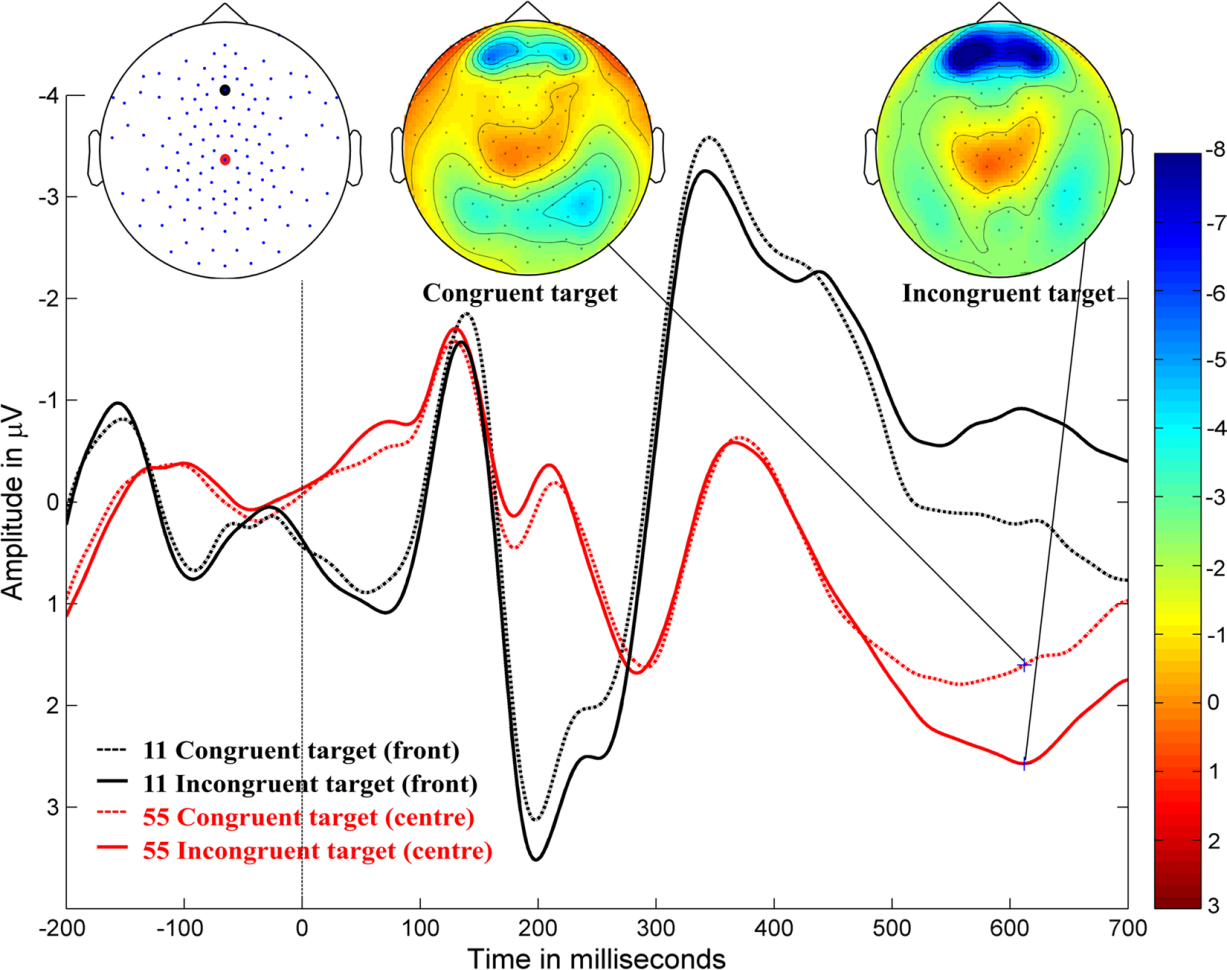
Inhibition. Grand-averaged ERP waveforms for congruent stimulus (dotted lines) and incongruent stimulus (solid lines) at the central electrode (55, red, behind Cz) and the frontal electrode (11, black, at Fz) in typically developing children (negativity up). Target stimulus onset is at 0 ms. Amplitude topographies for the congruent and incongruent target stimulus conditions at 612 ms after target stimulus onset.

The grand-averaged ERP waveforms and amplitude topographies for alerting, orienting, and inhibition in typically developing children are illustrated in Figs 1, 2, and 3, respectively.

**Figure 4:**
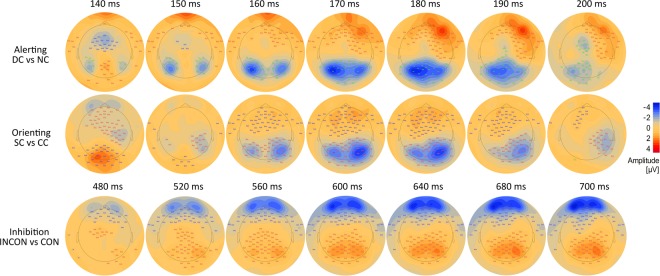
Cluster-based permutation tests for ERPs and amplitude difference topographies between conditions. Alerting: ERPs for the double-cued (DC) vs non-cued (NC) target stimulus (first row); orienting: ERPs for the spatially-cued (SC) vs centre-cued (CC) target stimulus (middle row); and inhibition: ERPs for the incongruent (INCON) vs congruent (CON) target stimulus (last row). Target stimulus onset is at 0 ms. Significant clusters were labelled with stars within the rectangles. ***p-values < 0.0005, **p-values between 0.005 and 0.0005. Blue and red colours indicate negative and positive amplitude values, respectively, from −4 to 4 µV.

Cluster-based permutation tests showed significant differences for each attention network contrast (Fig. 4). Significant alerting effects (*p* < 0.001) were presented in a negative cluster (i.e., the negative amplitude of double-cued target stimulus was larger than the negative amplitude of non-cued target stimulus) in the bilateral occipito-parietal areas, extending from approximately 140–200 ms following target stimulus onset, with a peak at approximately 170–180 ms; the difference between conditions was greater in the left hemisphere. There was also a negative cluster (*p* < 0.0001) over the central region from 140–150 ms, and a positive cluster (*p* < 0.0001) that originated in the frontal region and travelled to the right temporal region from 160–200 ms, with a greater difference in the right hemisphere.

A significant orienting effects (*p* < 0.0001) were evident in a negative cluster (i.e. the negative amplitude of the spatially-cued target stimulus was larger than the negative amplitude of the centre-cued target stimulus) in the bilateral occipital and right temporo-parietal areas, extending from approximately 150–200 ms after target stimulus onset, with a peak at approximately 160–180 ms; the difference between conditions was greater in the right hemisphere. The positive cluster (*p* < 0.0001) moved from the occipital to the mid-frontal area over the time range of 140–200 ms, with a greater difference in the left hemisphere.

Significant inhibition effects (*p* < 0.0001) were shown in a positive cluster (i.e. the positive amplitude of incongruent target stimulus was larger than the positive amplitude of congruent target stimulus) in the mid area and spread over the parietal and occipital areas, extending from approximately 480–700 ms after target stimulus onset, with a peak at approximately 560–640 ms; the difference between conditions was greater in the right hemisphere. Further, a negative cluster (*p* < 0.0001) was spread along the mid-frontal and left-central areas between 480 ms and 700 ms, with a greater difference in the left hemisphere. It is important to note that all these clusters did not have equal amplitudes across all time points.

### Neural source localization analysis

**Figure 5:**
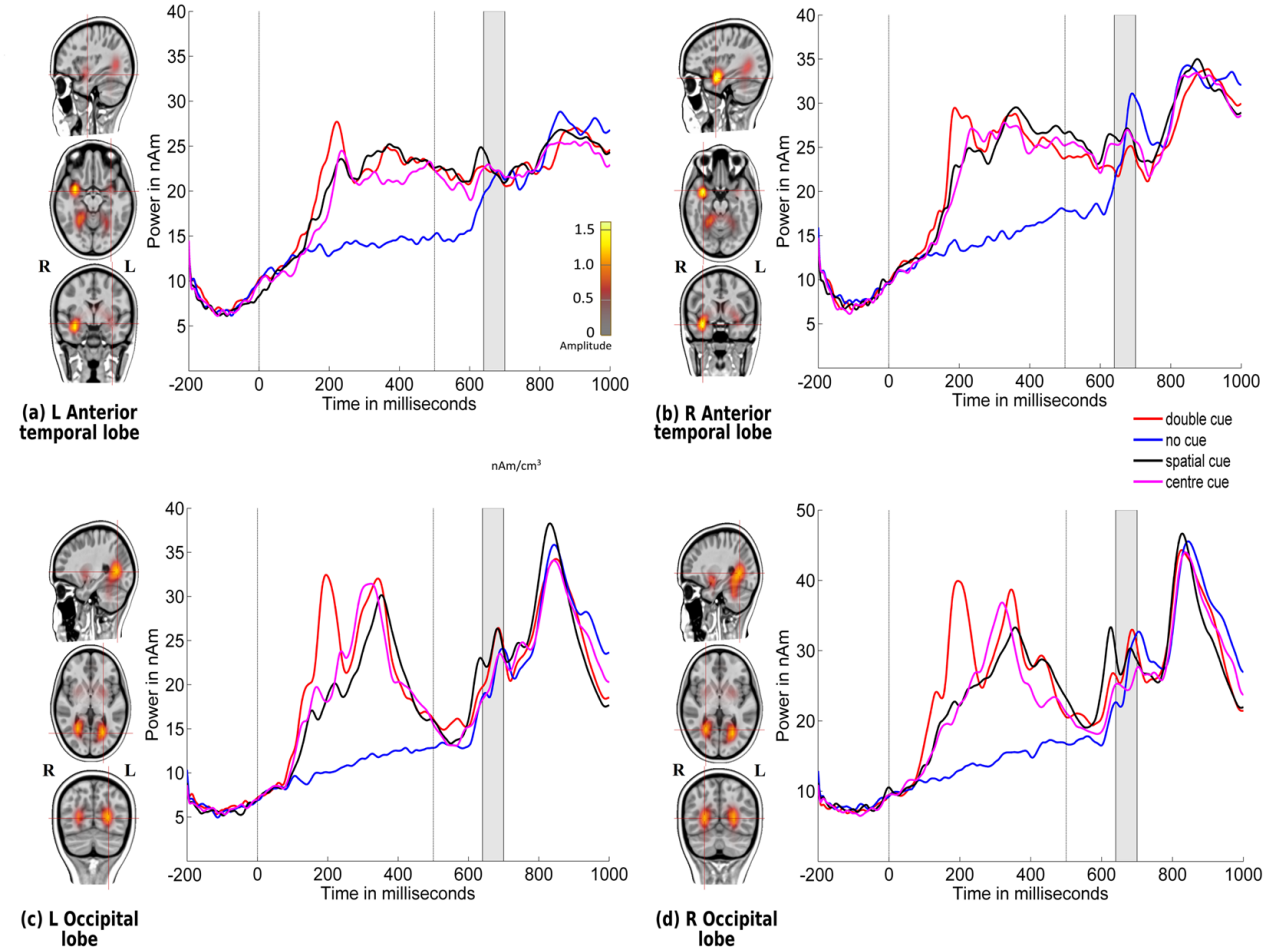
Source locations of grand-averaged ERPs collapsed across all conditions (congruent and incongruent stimuli: no cue, double cue, centre cue, and spatial cue) over time points of the N1 period of the target stimulus (140–200 ms) using CLARA in typically developing children. Cue onset is at 0 ms and target stimulus onset is at 500 ms. Brain activations were localized in the (**a**) left anterior temporal lobe, (**b**) right anterior temporal lobe, (**c**) left occipital lobe, and (**d**) right occipital lobe. Grand-averaged source waveforms for double-cued (red), non-cued (blue), spatially-cued (black), and centre-cued (magenta) stimuli, extracted using regional sources at the foci revealed by CLARA, are shown on the right side of each source. The colour bar denotes source amplitude. The shaded grey area denotes the source analysis time window.

Figure 5 illustrates the locations of source activations of grand-averaged ERPs collapsed across all conditions (congruent and incongruent stimuli with no cue, double cue, centre cue, and spatial cue) over the time points of the N1 period of the target stimulus (140–200 ms) using CLARA. Brain activations were found bilaterally in the anterior temporal and occipital lobes. Source-level statistics were calculated across individual participant source waveforms, based on the activation strength of the regional sources in the time window (140–200 ms after onset of target stimulus), thereby showing statistically significant effects at the electrode level. As shown in Table 2, for the alerting network, a significant effect was found in the left occipital lobe (*p* = 0.002) between 155 ms and 188 ms as well as in the right occipital lobe (*p* = 0.003) in the time window of 140–177 ms. The right anterior temporal lobe showed a significant response (*p* = 0.009) between 174 ms and 200 ms. For the orienting network, a significant effect was found in the left occipital lobe between 140 ms and 188 ms (*p* < 0.0001), and in the right occipital lobe from 162 ms to 193 ms (*p* = 0.006).

**Table 1:** Summary of the accuracy and reaction time (RT) results and statistics. Accuracy (mean and standard deviation) and RT (mean and standard deviation) of each stimulus condition of the ANT.

Conditions		Accuracy mean (standard deviation)	RT mean (standard deviation) in milliseconds	Paired t-test		
				M (SD)	t-value: df (82)	Cohen’s D_z_
Alerting	Non-cued	0.96 (0.03)	790 (99)	63.95 (40.26)	14.47***	1.59
	Double-cued	0.95 (0.04)	726 (85)			
Orienting	Centre-cued	0.96 (0.04)	762 (95)	54.31 (48.65)	10.17***	1.12
	Spatially-cued	0.96 (0.03)	707 (88)			
Inhibition	Incongruent	0.98 (0.02)	812 (98)	121.47 (51.85)	21.34***	2.35
	Congruent	0.94 (0.05)	690 (82)			

In the paired t-tests, M and SD denote the average difference and standard deviation of the difference between the RTs for two target stimuli, respectively. ***p < 0.0005 (two-tailed). The t-values denote test statistics with degrees of freedom (df) of 82. Cohen’s D_z_ denotes the effect size between RTs for different target stimuli.

**Table 2:** Talairach coordinates of sources during the N1 period of the target stimulus (140–200 ms).

Source	Brodmann area	Talairach coordinates (mm)			*p*-value (latency in ms)	
		X	Y	Z	Alerting (DC vs NC)	Orienting (SC vs CC)
L Anterior temporal lobe		−31.5	−2.9	−11.3	NS	NS
L Occipital lobe	BA19	−24.5	−65.9	2.7	= 0.002 (155–188)	< 0.0001 (140–188)
R Occipital lobe	BA19	24.5	−58.9	2.7	= 0.003 (140–177)	= 0.006 (162–193)
R Anterior temporal lobe	BA38	31.5	4.1	−18.3	= 0.009 (174–200)	NS

The table presents the *p*-values for alerting (double-cued vs non-cued stimuli) and orienting (spatially-cued vs centre-cued stimuli) sources and the latency of their effects. NS denotes not significant.

**Figure 6:**
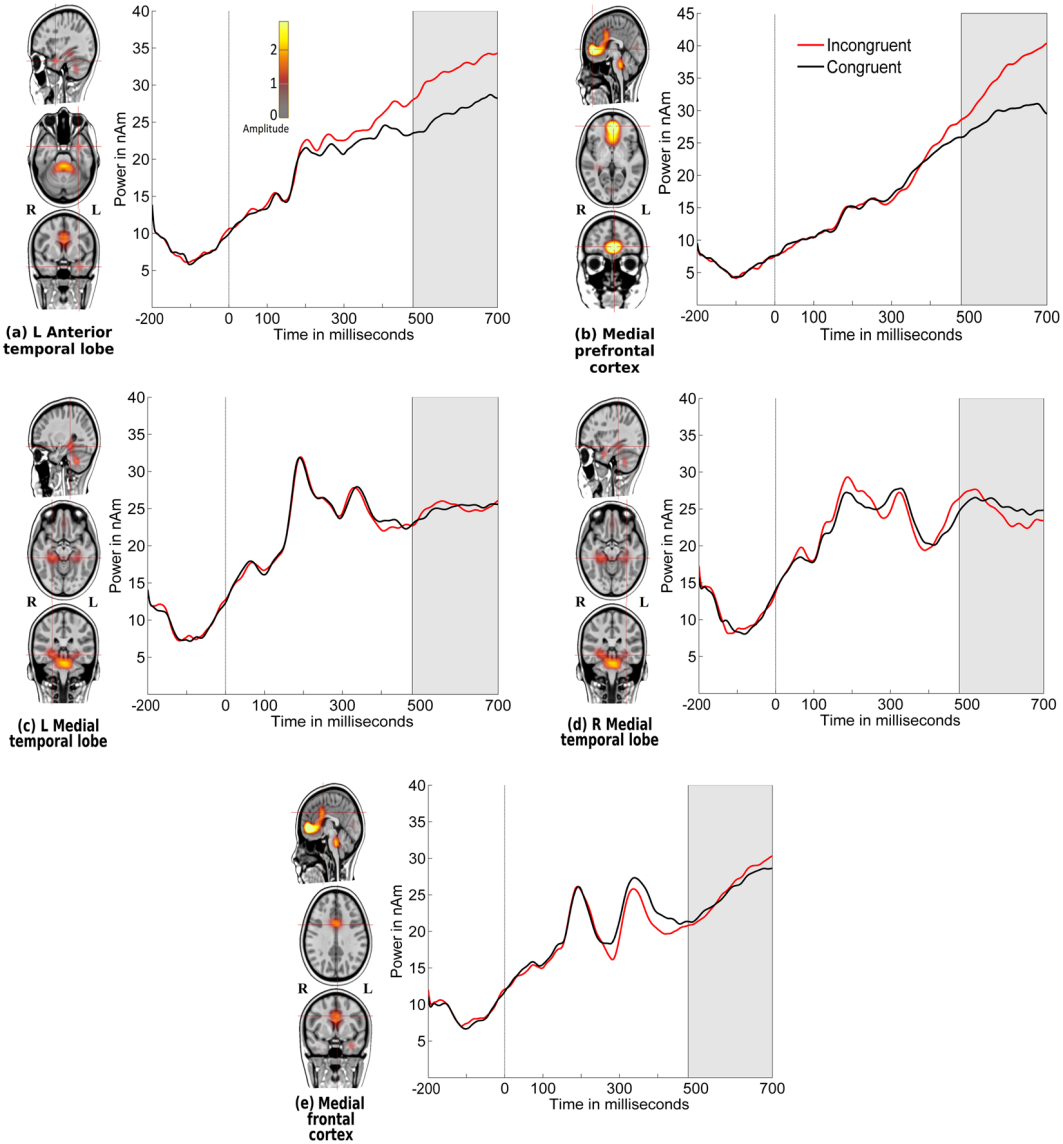
Source locations of grand-averaged ERPs collapsed across all conditions (congruent and incongruent stimuli with no cue, double cue, centre cue, and spatial cue) over time points of the target stimulus P3 period (480–700 ms) using CLARA. Target stimulus onset is at 0 ms. Brain activations were localized in the (**a**) left anterior temporal lobe, (**b**) medial prefrontal cortex, (**c**) left medial temporal lobe, (**d**) right medial temporal lobe, and (**e**) medial frontal cortex. The grand-averaged source waveforms for incongruent (red lines) and congruent (black lines) stimuli, extracted using regional sources at the foci revealed by CLARA, are shown on the right of each source. The colour bar denotes source amplitude. The shaded grey area denotes the source analysis time window.

Figure 6 illustrates the locations of source activations of grand-averaged ERPs collapsed across all conditions (congruent and incongruent stimuli with no cue, double cue, centre cue, and spatial cue) over the time points of the P3 period of the target stimulus (480–700 ms) using CLARA. Brain activations were found in the left anterior temporal lobe, medial prefrontal cortex, bilateral medial temporal lobe, and medial frontal cortex. As shown in Table 3, for the inhibition network, significant effects were found in the medial prefrontal cortex (*p* < 0.001) from 510 ms to 700 ms and in the left anterior temporal lobe (*p* < 0.0001) from 480 ms to 700 ms.

**Table 3:** Talairach coordinates of the sources for the target stimulus P3 period (480–700 ms)

Source	Brodmann area	Talairach coordinate (mm)			*p*-value (latency in ms)
		X	Y	Z	Inhibition (INCON vs CON)
Medial frontal cortex	BA24	−3.5	4.1	30.7	NS
Medial prefrontal cortex	BA32	−3.5	46.1	2.7	<0.001 (510–700)
L Anterior temporal lobe		−31.5	4.1	−25.3	<0.0001 (480–700)
L Medial temporal lobe	BA36	−31.5	−30.9	−11.3	NS
R Medial temporal lobe	BA36	24.5	−30.9	−11.3	NS

The table presents the *p*-values of the inhibition (incongruent vs congruent stimuli) sources and the latency of their effects. NS denotes not significant.

## Discussion

In this paper, we studied three attentional sub-processes derived from a modified version of the ANT for children. We examined (1) RT performance related to three attention networks: alerting, orienting, and inhibition in 12- and 13-year old typically developing children; (2) modulation of the visual N1 amplitude to the target stimulus by alerting and orienting sub-processes, and modulation of the P3 amplitude to the target stimulus by the inhibition sub-process; and (3) EEG-based neuronal sources related to these three sub-processes. We observed that visually cueing the stimulus enhanced performance; children responded faster in this case than in the absence of a cue. Further, we found that RTs to incongruent target stimuli were slower than those to congruent target stimuli. Alerting and orienting visual cues modulated the N1 amplitudes of target stimulus in the occipital and parietal areas. Further, P3 was modulated by target-to-flanker congruency across the centro-parietal and mid-frontal areas. Furthermore, EEG-based source-level statistics showed significant effects for alerting (double cue vs no cue) in the bilateral occipital lobe and right anterior temporal lobe, orienting (spatial cue vs centre cue) in the bilateral occipital lobe, and inhibition (incongruent vs congruent target stimuli) in the medial prefrontal cortex and left anterior temporal lobe.

The behavioural results of our study replicated those of existing research in both children^12,14,27,41^ and adults^5,9,21,27^ in terms of RT performance in the ANT. RTs to non-cued target stimuli were approximately 64 ms slower than RTs for double-cued target stimuli. Previous studies have suggested that alerting tends to improve from 10 years of age into adulthood^4,14,42^. RTs for spatially-cued target stimuli were approximately 55 ms faster than those for centrally-cued target stimuli. This shows that, like adults, children utilise orienting cues to extract the specific spatial location of an incoming target, and focus their spatial attention to the location prior to the onset of the target stimulus^12,14^. Our results are in line with previous studies, demonstrating that orienting tends to mature at an early stage of childhood^4,41,42^. The effect of inhibition manifested in faster RTs to a congruent target stimulus compared to an incongruent one, with a delay in response latency of approximately 122 ms for a target stimulus that is not aligned with its flankers. Overall, our results showed that cueing helps to enhance performance in children, while incongruence of the stimuli impedes performance, which follows Fan’s^9^ and Neuhaus’s^5^ findings on adults.

The effects of cueing and congruency manipulations were also reflected in the ERPs. Alerting and orienting effects were observed over occipital areas between 140–190 ms and 150–190 ms following target stimulus onset, respectively; whereas alerting effects in adults have shown centro-parietal topography^5^. Further, we found that orienting effects in children align with prior research in terms of effect timing and topographic differences in adults^5^. Inhibition effects were observed at 520–700 ms in the centro-parietal areas. The topographic differences in our study demonstrated a reversed polarity to that from a previous study on adults^5^, likely because of a higher P3 amplitude for incongruent stimuli in children, which has also been demonstrated in other studies^6,39^. This developmental effect is further discussed below.

In our study, the modulation of the visual N1 amplitude to the target stimulus by alerting and orienting cues in children was consistent with prior findings in adults^5,16,27^. However, no previous studies examine a cueing effect on the visual N1 for the target stimulus with flanker stimuli in children. Our finding of enhanced N1 for the double-cued target stimulus in the alerting sub-process may indicate more efficient processing of the target stimulus following specific warning cues (double cue). Similar to alerting, orienting manipulation enhanced the N1 when spatially cued. This again reflected enhanced processing of the target stimulus, with attention allocation to the correct spatial location.

As a final stage of the analysis, we examined our source-level information to disentangle possible neural sources beyond the scalp potentials. The N1-related sources of the target stimulus were localized to the right anterior temporal lobe, left and right occipital lobes, and left anterior temporal lobe. We excluded deep sources (pons, cerebellum) because these sources make a smaller contribution to the scalp recording of ERPs than cortical sources^43,44^; therefore, they can be less reliable.

For the alerting network, a significant effect was shown across multiple neuronal sources between 140 ms and 200 ms. These sources were the left and right occipital lobes and the right anterior temporal lobe. Our results showed activation in both hemispheres for alerting and orienting networks. The activity in the left and right occipital lobes could be interpreted as enhanced visual processing of a target stimulus appearing in the cued location^45–47^. Modulation of activity in the right anterior temporal lobe, which has been found in previous fMRI studies^13,22^ and in other experiments^48^ on intrinsic alertness using PET data, replicated in our findings in more anterior temporal areas. This region is generally thought to be involved in the facilitation of alertness in the brain in response to warning cues^49,50^. The lack of an alerting effect in the activity of frontal and parietal areas in children is discussed below, along with the orienting effect.

For orienting, a significant effect was shown in the bilateral occipital lobe between 140 ms and 200 ms. Bilateral occipital lobe activity was shown to be modulated by gaze shifts and direction of eye movement over the target stimulus and its flankers^47^. Our findings regarding the orienting network in children are in accordance with functional neuroimaging studies on children^13^ and orienting effects in adults^23^ that relate to the activation of the occipital lobe. In this study, similar to alerting, the orienting network showed reduced or absence of frontal and parietal activation in children. Prior studies have shown that the adult attention network utilises frontal-parietal areas to maintain a state of readiness for processing of non-specific cueing target information^22,51^. Thus, it appears that this maintenance of readiness matures only in late childhood, after 12 years of age, and fronto-parietal activity in adults might reflect more top-down control of attention that is not utilised by children in the ANT^37^. On the other hand, in previous studies on adults that employed the ANT, the experimental design differs from that in the current study in a number of ways: longer cue-to-target interval and random variation of the duration of this interval^22^, appearance of the target on either the left or right of a fixation cross^13^, and size and shape of the target stimulus^21^. Previous studies have suggested that these specific parameters might have an effect on task demands and network effects^29,42^. Further, in our study, there was no significant correlation between observed RT performance and neuronal source strength. Along with the finding of expected RT pattern, this suggests that RT performance reflects the sum of differences in the processes produced by RT performance, and that the processes measured in this study might be sensitive only to a few of the attributes of cognition that mediate task performance^13,52^.

Further, we found that the modulation of the P3 amplitude to the target stimulus associated with the inhibition response in children had significant effects in the centro-parietal and mid-frontal areas. P3 amplitudes for incongruent target stimuli at the centro-parietal electrodes were considerably higher than for congruent target stimuli. This increase in the P3 amplitudes of incongruent target stimuli in children is consistent with previous ANT studies on children^6,15,39^. A possible interpretation could be higher discriminability of the target stimulus from its flankers, which led to a higher P3 amplitude in the centro-parietal areas^53^. However, ANT studies on young adults show decreased amplitudes in response to incongruent versus congruent targets for the P3 component^5,21^. This discrepancy between the results for children and adults might be better interpreted based on the source analysis.

For inhibition, source-level statistics showed significant activation in the medial prefrontal cortex and left anterior temporal lobe. A large number of neuroimaging studies have revealed that the prefrontal cortex (ACC) is involved in the inhibition network in both children^13,15^ and adults^22,54–56^. Evidence from a previous fMRI study on children showed that the left temporal lobe is strongly associated with response inhibition^34^, although our source localization was more anterior. The increased ERP amplitude to incongruent versus congruent target stimuli is in line with source analysis results and with the involvement of the ACC region in conflict resolution^4,22^. The lack of modulation of other cortical regions by the congruency of the target stimulus (e.g. the fusiform gyri^22^) might explain the different direction of the P3 effect between children and adults.

In a developmental study of attention networks in children and adults, significant lateralization differences were found in the temporal lobe (superior temporal gyrus)^13^. One explanation for activation of this area in children relates to their verbal strategies^34^. However, it is challenging to define a single function for the temporal lobe or prefrontal cortex, because they include higher-order cortical regions (i.e. superior temporal gyrus and ACC) that are involved in several cognitive processes^57^. However, our study suggests that these areas also play an important role in children’s inhibition networks.

Overall, our study has certain limitations. The first is the fact that current EEG/ERP source imaging is an estimation of brain activity with rather limited spatial resolution. The distributed source imaging could produce more source activation spread^58,59^, which would make it difficult to distinguish between close hemispheric areas and specific brain voxels or regions. Second, we used a child template MRI rather than individual MRIs for our participants, which likely resulted in some loss of precision in source imaging^60^. However, this method has been used in previous source analysis studies and remains a better option than using adult templates for studies on children^61,62^. Further, by examining the spatial correspondence between high-density EEG/ERP source localization and fMRI activation in children^63,64^ and individual level MRI-constrained EEG source localization^65^ could help to more precise source localization.

In summary, our study demonstrated that children replicated classic attentional behavioural RT performances using a modified version of the ANT. Our study shows significant modulation of the visual N1 amplitude to a target stimulus by the alerting and orienting sub-processes of attention, and modulation of the P3 amplitude to the target stimulus by the inhibition sub-process. However, despite their classic behavioural performance pattern, ERP and source results for children are different from those for adults. Thus, our results indicate reduced top-down control mechanisms in children for the alerting and orienting sub-processes, evidenced by a lack of fronto-parietal network activation, which could at least partially be explained by differences in fMRI and EEG experimental designs. The combined study of RTs, ERPs, and their neuronal sources provides a comprehensive view of the mechanisms that underlie attentional networks in children. Future studies could extend our findings to explore attentional processes in children with attentional problems and other developmental difficulties.

## Methods

### Participants

Eighty-three Finnish children aged between 12 and 13 years (43 girls, 40 boys; mean age 12.38 years, SD: 0.48) and studying in the sixth grade participated in this study. All children had normal or corrected vision, with no history of neurological problems or head injuries. They were recruited to the eSeek project (Internet and Learning Difficulties: A Multidisciplinary Approach for Understanding Reading in New Media). The study was conducted in compliance with the Declaration of Helsinki, and study protocols were approved by the ethics committee of the University of Jyväskylä, Finland. All methods were performed in accordance with the relevant guidelines and regulations. The participants and their parents provided signed informed consent prior to the study. The analysed data sets from this study are available from the research group upon request.

### Procedure: Attention Network Test for Children

In this study, we used the modified version of the ANT^5^ to measure the three sub-components of the attention network: alerting, orienting, and inhibition. Participants were required to lean on a chinrest located 60 cm from a 24-inch computer screen (resolution of 1920 × 1080 and a refresh rate of 60 Hz). A fixation cross was visible in the centre of the white-colour screen (960, 540 (x, y)) during the entire testing period (i.e. not during eye-tracker calibration). The participant’s task was to look at the fixation cross and report the direction of the middle fish as quickly and accurately as possible by pressing a corresponding button.

**Figure 7:**
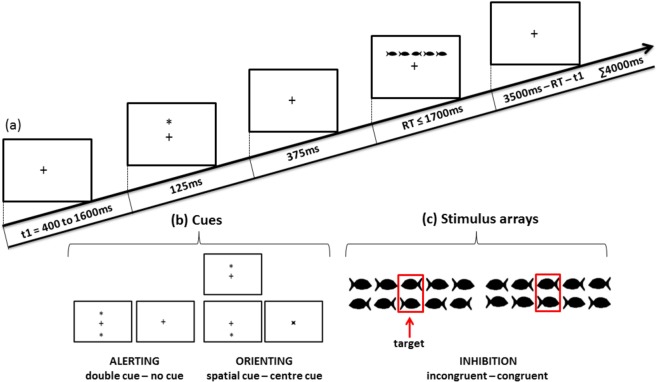
(**a**) Schematic illustration of the sequence of events in the modified ANT; t1 denotes a fixation period of a random duration between 400 and 1600 ms, (**b**) the four cue conditions used in ANT, and (**c**) the two congruency conditions for which the children had to decide the swimming direction of the middle fish.

As shown in Fig. 7, the stimulus (a group of fish) was preceded by one of the four cue conditions (no cue, double cue, centre cue, or spatial cue). The fixation period of a random duration was between 400 ms and 1600 ms before cue appearing. The duration of the cue was 125 ms, which was followed by 375 ms of waiting time before the stimulus was presented (a total of 500 ms prior to stimulus presentation). In the double cue trial, two asterisks were presented simultaneously at a 1° angle above and below the fixation cross. In the centre cue trial, an asterisk was presented on the fixation cross. In the spatial cue trial, a single asterisk appeared in the position of the upcoming stimulus.

To make the experiment more child-friendly, black fish drawings were used as stimulus. The stimulus comprised a row of five horizontal fish. Each fish was subtended to 0.7°, and adjacent fish were separated by 0.3° each. The size of the entire stimulus array was 4.7°. The centre fish in the stimulus was the target, and the two fish on either side of the target were referred to as flankers. The stimulus array in each trial was presented above or below the fixation cross, at the same location where the double cue or spatial cue appeared. The maximum duration of each trial was 4000 ms. The maximum duration of the stimulus array in each trial was 1700 ms, until a response was detected; thereafter, if there was no response, it was considered an unattended trial and terminated. The maximum duration between the offset of the stimulus and the start time of the next trial was 3500 ms, which varied according to the duration of the stimulus array. For congruent stimuli, the flankers were in the same direction as the target and for incongruent stimuli, the flankers were in the opposite direction. Participants were instructed to keep their gaze on the fixation cross throughout the experiment and report the swimming direction of the centre fish by pressing a left or right corresponding direction button in the button box.

One ANT session consisted of 288 pseudo-randomized trials, which were divided into 4 experimental blocks, with 72 trials in each block. Each block consisted of all eight possible conditions in equal proportions: four cue conditions (no cue, double cue, centre cue, and spatial cue) × two stimulus conditions (congruent, incongruent).

### EEG and eye-tracker recording

The experiment was designed using the Experiment Builder (1.10.1630) software on a Dell Precision T5500 workstation. EEG data were recorded using a high-density array of 128 Ag-AgCl electrodes in HydroCel Geodesic Sensor Nets (Electrical Geodesics Inc.). The EEG was amplified using a NeurOne amplifier (Mega Electronics Ltd.). During measurement, the impedance of most electrodes was kept below 50 kΩ, and the quality of the EEG data was monitored throughout the EEG recording. EEG was referenced to Cz online and sampled at 1000 Hz. An online high-pass filter of 0.16 Hz and low-pass filter of 250 Hz were applied during EEG data recording. Further, eye movement data were recorded with a table-mounted Eyelink 1000 eye tracking device at 1000 Hz for both eyes (SR Research Ltd.). EEG and eye movements were recorded simultaneously through the combination of triggering via Ethernet messages and TTL pulses. The entire experiment was conducted in a dimly lit sound-attenuated room in a laboratory at the University of Jyväskylä, Finland.

### Pre-processing of EEG data and eye tracking

EEG data were pre-processed using MatLab R2014a, with toolboxes EEGLab^66^ (Swartz Centre for Computational Neuroscience, San Diego) and FieldTrip^67^ (version 20160110), and BESA Research 6.1 (BESA GmbH, Munich, Germany). The raw eye-tracking data were converted and stored into a MatLab-structured array using the EEGLab add-on EYE-EEG^68^. The continuous raw EEG data file was imported into EEGLab, in which bad channels were interpolated and the EEG was synchronized with the eye-tracking data. The quality of synchronization was checked by comparing eye-tracking event latencies against EEG event latencies.

A high-pass filter of 0.5 Hz (fifth order, zero-phase Butterworth filter) was applied to the raw EEG data. It was segmented into 1200 ms (200 ms before cue onset and 1000 ms after cue onset) for non-cued, double-cued, centre-cued, and spatially-cued stimuli, and 900 ms (200 ms before and 700 ms after the onset of target stimulus) for congruent and incongruent stimuli. Then, a low-pass filter of 30 Hz (sixth order, zero-phase Butterworth filter) was applied to the high-pass filtered, segmented EEG data (trials). The baseline was set to −200 ms and 0 ms of the filtered segmented data. Gaze positions in each trial were examined in order to ensure that participants maintained their gaze in the optimal position for stimulus presentation. As such, if there was an eye blink, the gaze position value was recorded as zero, which was outside the defined area (860–1060, 440–640 (x, y)) on the display screen; such trials were eliminated. Trials with muscular and other artefacts were rejected using a threshold rejection approach. Moreover, trials with a difference between the maximum and minimum voltages within an ERP epoch that exceeded 175 µV were rejected prior to calculating the average. Accepted trials using the above criteria were averaged for each participant. The averaged ERPs were re-referenced to average reference in BESA Research 6.1 (BESA GmbH, Munich, Germany). Each condition (non-cued, double-cued, centre-cued, spatially-cued, congruent, and incongruent) had a minimum of 30 trials for averaging. The average number of correctly responded trials for both cued (double, centre, and spatial) and non-cued conditions were 65; the average number of correctly responded trials for incongruent and congruent target stimuli were 134 and 128, respectively. The EEG data of 12 participants were excluded from the analysis because of eye blinks (11 participants) and physical movement artefacts (1 participant).

### Data analysis

#### Statistical analysis of RT data

The RTs of each trial were calculated from target stimulus onset time to button response time. Unattended trials, trials with incorrect responses, and trials which were not accepted for ERP averaging were excluded from calculations of the mean RTs. All participants maintained a high level of accuracy (see Table 1). Paired-sample t-tests were performed in IBM SPSS Statistics Version 24 to determine significant differences in RTs between conditions. There were no outliers, and the data was approximately normally distributed.

### Statistical analysis of the EEG and eye-tracking data

Nonparametric, cluster-based permutation t-tests were performed as a two-tailed test in BESA Statistics 1.0 (BESA GmbH, Munich, Germany) for revealing significant effects across all the electrodes in alerting (double-cued vs non-cued target stimulus), orienting (spatially-cued vs centre-cued stimulus), and inhibition (incongruent vs congruent target stimulus). For alerting and orienting conditions, ERP statistics were calculated between 500 ms (the onset of target stimulus) and 1000 ms from the onset of the cue. For the inhibition condition, ERP statistics were calculated from the onset of the target stimulus to 700 ms. The number of permutations was set to 1000, and cluster alpha (the significance threshold level for data to enter a cluster) was set to 0.05. For spatial clustering, the neighbour distance between electrodes was set to 3 cm. (Permutation testing is a non-parametric statistical approach that uses the arbitrary division of groups or conditions to evaluate the distribution of the effect of interest. By comparing the original test statistic with the permutation distribution, it is possible to compute a *p*-value for the effect^69–71^).

### Source analysis

Source analysis was performed in BESA Research 6.1 to estimate source areas in the brain that were responsible for the alerting, orienting, and inhibition sub-components of attention. The source areas associated with alerting were calculated by the difference in response between double-cued and non-cued target stimuli, those in orienting by the difference between spatially-cued and centre-cued target stimuli, and those in inhibition by the difference between incongruent and congruent target stimuli. The distributed source model CLARA^40^ was used to localize neuronal sources. For effective forward-head modelling, an age-appropriate FEM head model for 12-year-olds implemented in BESA Research 6.1 was selected. The time window of interest for the N1 period of the target stimulus was between 140 ms and 200 ms, and the time window for the P3 period of the target stimulus was between 480 ms and 700 ms. Source locations were calculated for N1 and P3 target stimuli periods of the grand-averaged ERPs, which were collapsed across all the conditions (congruent and incongruent conditions for no cue, double cue, centre cue, and spatial cue). The source analysis time interval was selected on the basis of the significant time window from the cluster-based permutation tests of the ERPs. A regional source was considered as three single dipoles at the same location, with three orthogonal orientations^40^. This source was fitted in the foci obtained from the CLARA solution. The source strength at each time point was estimated as a combined sum of the power of the three orthogonal orientations of the regional sources. These regional sources were used as a spatial filter for source modelling for each of the three effects. Source-level paired t-test statistics were calculated using a two-tailed test based on the individual-level source waveforms associated with the locations of the neuronal sources obtained from N1 and P3 periods of the collapsed grand-averaged ERP between conditions.
